# Fabrication of Silk Hydrogel Scaffolds with Aligned Porous Structures and Tunable Mechanical Properties

**DOI:** 10.3390/gels9030181

**Published:** 2023-02-24

**Authors:** Zewu Jiang, Qingqing Sun, Qian Li, Xiaomeng Li

**Affiliations:** 1 School of Mechanics and Safety Engineering, Zhengzhou University, Zhengzhou 450001, China; 2 National Center for International Joint Research of Micro-Nano Moulding Technology, Zhengzhou University, Zhengzhou 450001, China; 3 School of Materials Science and Engineering, Zhengzhou University, Zhengzhou 450001, China

**Keywords:** silk hydrogel scaffold, mechanical property, aligned porous structure, freeze-casting, photo-crosslinking, tissue engineering

## Abstract

The effectiveness of cell culture and tissue regeneration largely depends on the structural and physiochemical characteristics of tissue-engineering scaffolds. Hydrogels are frequently employed in tissue engineering because of their high-water content and strong biocompatibility, making them the ideal scaffold materials for simulating tissue structures and properties. However, hydrogels created using traditional methods have low mechanical strength and a non-porous structure, which severely restrict their application. Herein, we successfully developed silk fibroin glycidyl methacrylate (SF-GMA) hydrogels with oriented porous structures and substantial toughness through directional freezing (DF) and in situ photo-crosslinking (DF-SF-GMA). The oriented porous structures in the DF-SF-GMA hydrogels were induced by directional ice templates and maintained after photo-crosslinking. The mechanical properties, particularly the toughness, of these scaffolds were enhanced compared to the traditional bulk hydrogels. Interestingly, the DF-SF-GMA hydrogels exhibit fast stress relaxation and variable viscoelasticity. The remarkable biocompatibility of the DF-SF-GMA hydrogels was further demonstrated in cell culture. Accordingly, this work reports a method to prepare tough SF hydrogels with aligned porous structures, which can be extensively applied to cell culture and tissue engineering.

## 1. Introduction

Tissue-engineering (TE) scaffold primarily provides the seeding cells with specific microenvironments to guide cell proliferation and differentiation and, thereby, enhances tissue regeneration [1,2,3]. Hydrogels have gained increasing attention in the field of TE due to their resemblance to the cellular microenvironment in vivo and tunable physicochemical features. Hydrogels based on natural materials, such as alginate [4], hyaluronic acid [5], gelatin [6], and silk fibroin (SF) [7], can exhibit good biocompatibility and biodegradability [8,9]. Through mild crosslinking and processing techniques, hydrogels with diverse shapes, structures, and functions can be created for biomedical applications [10].

SF has been widely used in biomedical fields, such as cell culture [11], drug delivery [12,13], and tissue engineering [14], due to its high biocompatibility, degradability, and absorbability [15], as well as the fact that it has been approved by the US Food and Drug Administration (FDA) [16]. Physical crosslinking is typically utilized to transform the random coil into the β-sheet conformation while fabricating a SF hydrogel for TE [17]. However, this process is difficult to control, and the mechanical properties of the prepared SF scaffolds frequently fail to fulfill the criteria [18]. *N*-(3-Dimethylaminopropyl)-*N*’-ethyl carbodiimide hydrochloride (EDC), *N*-Hydroxysuccinimide (NHS), and other crosslinking agents are frequently used for chemical crosslinking to form amide bonds [19,20]. Nevertheless, the residual crosslinking agents may have an impact on cell viability. SF hydrogels with tunable mechanical properties have recently been enzymatically crosslinked using horseradish peroxidase for encapsulating human ovarian stromal cells [21]. Similarly, the cascade enzymatic crosslinking approach is employed to make functional SF hydrogels with a three-dimensional porous microenvironment for wound healing [22]. However, the displayed 3D porous structure is formed prior to observation by freeze drying. SF hydrogels with stable mechanical properties and low β-sheet content are crosslinked through di-tyrosine bonds by visible light, which can support the long-term culture of chondrocytes [23]. Meanwhile, photo-crosslinked silk fibroin glycidyl methacrylate (SF-GMA) hydrogels have been extensively applied in biomedical engineering due to their mild reaction conditions, few by-products, easy control of the reaction process, and high reaction efficiency [24,25]. However, the non-porous structure, brittle mechanical properties, and low toughness of these SF hydrogel scaffolds limit their further applications [26,27,28].

Porous hydrogel scaffolds have been shown to enhance nutrient diffusion and cell migration and proliferation [29,30]. Scaffolds with aligned porous structures can further guide cell morphology and promote tissue regeneration [31,32]. There are numerous strategies for controlling the porosity and microarchitecture of TE hydrogels [33]. In particular, freeze casting can be utilized to regulate the microscopic molecular arrangement of such gels and control pore morphology by controlling the directional ice formation in the solute solution [34]. Ice crystals form and propagate through predetermined directions during freezing, and melted ice crystals form interconnected microchannels within the scaffold [35]. Using ice templates to create programmable microporous structures eliminates the need for organic solvents [36,37]. For example, polyvinyl alcohol (PVA) hydrogels with high strength, toughness, and fatigue resistance were generated using directional freezing and salting-out treatment [38]. Bai et al., adjusted the PDMS wedges on the surface of a cold source to control the rate of ice crystal formation in the horizontal and vertical directions and produce large-scale aligned porous materials [39]. Additionally, using this technique, the degradable silk nacre and controllable performance were successfully processed [40]. However, the porous scaffolds prepared by current freeze-casting technology generally maintain a microporous structure after the freeze-drying and crosslinking treatment [41,42], and the obtained materials are unsuitable for 3D cell encapsulation and soft tissue engineering.

**Figure 1:**
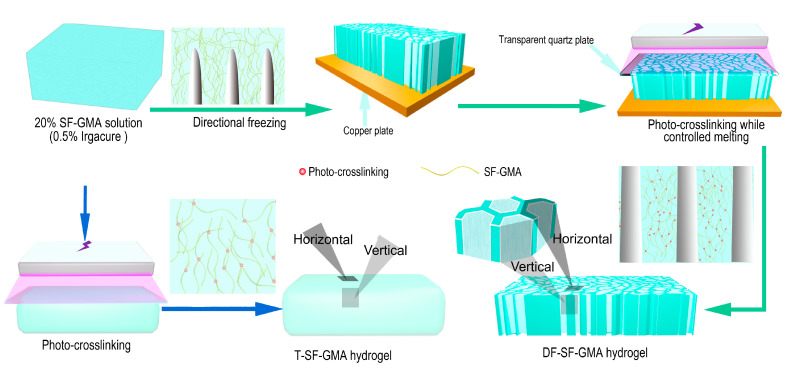
The schematic diagram for the fabrication of the glycidyl methacrylate (GMA) modified silk fibroin (SF) hydrogels with aligned porous structures via directional freezing (DF) and photo-crosslinking (DF-SF-GMA hydrogel) and the traditional SF hydrogel (T-SF-GMA hydrogel). The aligned porous structure of the DF-SF-GMA hydrogels is templated from ice crystal formation due to DF and is maintained after photo-crosslinking during controlled melting.

Here, we combine freeze-casting and photopolymerization processes to develop a silk hydrogel scaffold with aligned porous structures and robust mechanical properties. As illustrated schematically (Figure 1), a silk fibroin glycidyl methacrylate (SF-GMA) hydrogel with an aligned porous structure was prepared after directional freezing and UV crosslinking during the melting process. The oriented interconnected structures formed by the growth of ice crystals can be observed through an SEM. The mechanical properties of this directional-freezing and photo-crosslinked SF-GMA scaffold (DF-SF-GMA) were thoroughly investigated. The enhanced mechanical strength and tunable viscoelasticity are achieved due to the aligned porous structure. In addition, the secondary structure of the silk fibroin during this process was explored. The toughness of the DF-SF-GMA hydrogels is improved due to the porous structure and the alteration of the secondary structure. Then, the cytotoxicity of the DF-SF-GMA hydrogels was also examined to assess the potential for tissue regeneration applications.

## 2. Results and Discussion

### 2.1. SF-GMA Characterization and Hydrogel Secondary Structure

**Figure 2:**
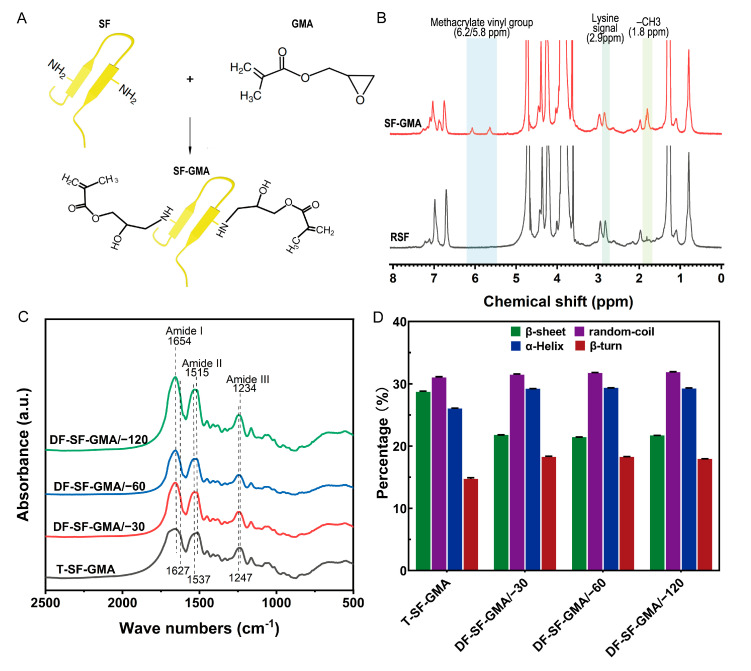
FTIR spectra and secondary structures of hydrogels. (**A**) Modification of SF molecule with GMA. SF was covalently grafted with GMA, which provides a vinyl double bond as a UV-crosslinking site. (**B**) ^1^H-NMR spectra of the pristine RSF and the SF-GMA. The modification of lysine residues in SF with GMA was confirmed by the gradual decrease in the lysine signal and an increase in the methacrylate vinyl group and the methyl group signals. (**C**) FTIR spectra of the T-SF-GMA and DF-SF-GMA hydrogels. (**D**) Quantitative analysis of secondary structures in the T-SF-GMA and DF-SF-GMA hydrogels. Data are shown as mean ± S.D.; *n* = 3.

The SF-GMA hydrogel was synthesized by methacrylate substitution of the SF primary amines (Figure 2A). To create a SF-GMA hydrogel with a high degree of functionalization, excess GMA was added to the regenerated SF solution. The success of grafting through the reaction of ring-opening of epoxy at the GMA and amino group on the molecular chain of SF was demonstrated using a proton nuclear magnetic resonance (^1^H-NMR) spectroscopy (Figure 2B). It is evident that a characteristic resonance of the methacrylate vinyl group at δ = 5.8–6.2 ppm and a characteristic resonance of the methyl group of GMA at δ = 1.8 ppm appear in the SF-GMA macromer group. Moreover, there was a slight decrease in the lysine methylene signal at δ = 2.9 ppm after GMA modification, which all indicate the successful synthesis of the SF-GMA. The functionalization degree was calculated using the ^1^H-NMR spectra, and the methacrylate degree of the SF-GMA is 36.4%.

The FTIR analysis was used to evaluate the secondary structures of the SF hydrogels (Figure 2C). The FTIR spectral regions between 1700–1600 cm^−1^ and 1600–1500 cm^−1^ represent the peptide backbone absorptions of amide I and amide II, respectively [43]. It indicates that all groups have a strong peak at 1654 cm^−1^, which is assigned to the α-helix conformation. A prominent shoulder peak at 1627 cm^−1^ and a peak at 1515 cm^−1^ in amide II correspond to the β-sheet conformation that appears in the T-SF-GMA group, but it is not visible in the DF-SF-GMA group [44]. There is a strong peak at 1537 cm^−1^ in the directional-freezing groups, which is assigned to the random coil conformation [45].

To quantitatively analyze the content of the secondary structure of the SF-GMA hydrogels, the amide I region (1700–1600 cm^−1^) in the FTIR spectra of the SF-GMA hydrogels was examined through Fourier self-deconvolution (FSD) (Figure S1A,B). The deconvolution of these spectra reveals 28.6% β-sheet, about 30.9% random coil, 25.9% α-helix, and 14.6% β-turn in the T-SF-GMA hydrogel (Figure 2D). Among the DF-SF-GMA groups, the content of their secondary structure is basically the same. Compared to the T group, the β-sheet content of the DF-SF-GMA groups decreases by about 7%, and the random coil content remains virtually unchanged, while the α-helix content and β-turn content increase by 3.3% and 3.6%, respectively. Importantly, these results show that the transformation from random coil to β-sheet structures is prevented during directional freezing and photo-crosslinking. It has been reported that a reduction in β-sheet normally improves the toughness of SF materials [46]. Similarly, the reduced β-sheet content caused by directional freezing enhances the toughness of the DF-SF-GMA hydrogels.

### 2.2. The Porous Architecture

**Figure 3:**
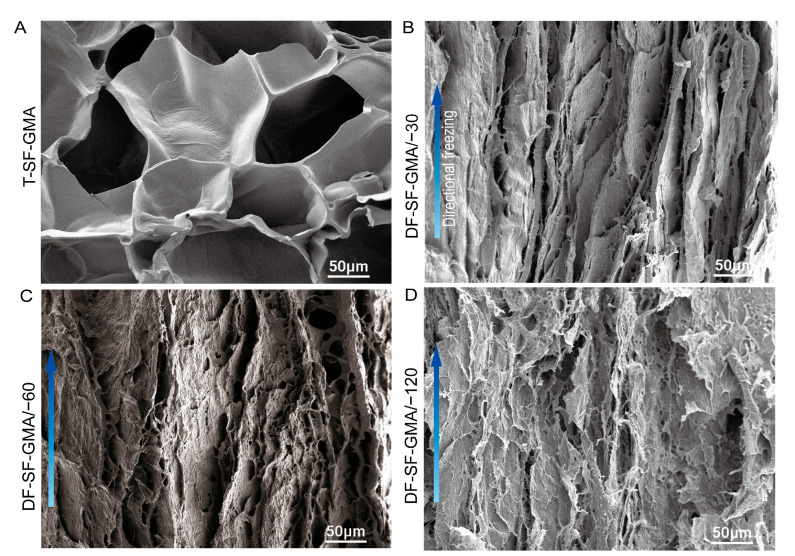
Characterization of the aligned porous structures of the DF-SF-GMA hydrogels. Scanning electron microscopy (SEM) images of the vertical section of the traditional hydrogel (T-SF-GMA) (**A**) and the hydrogels fabricated by directional freezing at −30 °C (DF-SF-GMA/−30) (**B**), −60 °C (DF-SF-GMA/−60) (**C**), and −120 °C (DF-SF-GMA/−120) (**D**). The porous structures in the T-SF-GMA hydrogel are induced by ice particles randomly formed during freezing. The SF molecules in the DF-SF-GMA hydrogels are excluded from the ice and aggregate between the aligned ice crystals and photo-crosslinked during melting. These aligned porous structures already exist prior to freeze drying.

The aligned porous lamellar structure in the SF hydrogels was constructed by the ice templates induced by directional freezing. As shown in Figure 3, the DF-SF-GMA hydrogels have a lamellar structure along the ice crystal growth direction. Moreover, the distance between the layers is larger as the freezing temperature increases. The hydrogel generated in the −30 °C plate exhibits the largest pore size, indicating less initial nucleation and a slower growth rate of ice crystals [47]. The morphology and structure of the control T-SF-GMA hydrogel are basically consistent with traditional bulk hydrogels [48]. The homogeneous pores in this hydrogel are formed by randomly growing ice crystals.

### 2.3. Mechanical Properties of SF-GMA

**Figure 4:**
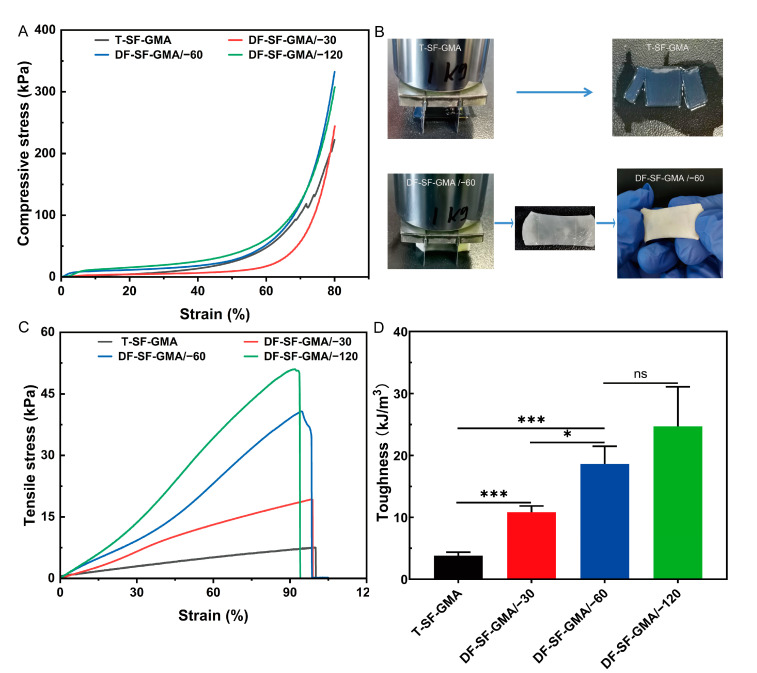
Physical properties of the DF-SF-GMA hydrogels. (**A**) Compression test for the T-SF-GMA and DF-SF-GMA hydrogels with varied freezing temperatures. (**B**) The T-SF-GMA and DF-SF-GMA/−60 were cut and compressed by a 1 kg weight for 1 min. The DF-SF-GMA/−60 hydrogel was still intact after removing the weight. (**C**) Tensile test for the SF-GMA hydrogels. (**D**) Toughness of the T-SF-GMA and DF-SF-GMA hydrogels (* *p* < 0.05; *** *p* < 0.001, *n* = 3).

The brittleness of traditional hydrogels is an inherent challenge in tissue engineering due to the architecture of hydrogels. DF-SF-GMA hydrogels with directional porous structures were fabricated in this study. The compression curves and the compressive modulus of these hydrogels were tested and determined to compare the different mechanical behaviors of the hydrogels with varied structures. The stress–strain curves show that the stress in the directional-freezing groups increases dramatically in the first 10% region (Figure 4A and Figure S2A), which may be attributed to the supporting of the directional lamellar structure formed during the ice crystal growth. Furthermore, the reinforcement in the directional-freezing hydrogel differs depending on the freezing temperatures, and the stress increases greater at a lower temperature. However, the stress of the control group increases gradually only from 0 kPa to around 1.7 kPa throughout the 10% strain.

In the control group, the stress drops vertically at a strain of 72% (Figure 4A and Figure S2B), indicating a crack in the hydrogel. However, the DF-SF-GMA groups remain intact after compression. These results indicate that the toughness of SF hydrogels can be enhanced via directional freezing and photo-crosslinking, which may benefit from the oriented layered structure and the reduced content of β-sheet.

The compressive modulus of group T increases gradually at 3%, 25%, and 50% strain points (Figure S2C), which is consistent with the compressive properties of traditional photo-crosslinked hydrogels. Interestingly, the directional-freezing hydrogels show distinct characteristics. The compressive modulus of the DF-SF-GMA hydrogels produced at −60 °C and −120 °C shows a considerable enhancement at the strain of 3%, and then it falls at 25% and gradually increases again at 50%. These compressive behaviors are probably because of buckling. The lamellar materials formed by directional freezing play a supporting role in the early stage, buckling occurs as the strain increases, and then the modulus increases further with the densification of the materials.

Traditional hydrogels are not only easily fractured and brittle under pressure, but they are also incapable of stretching. The tensile mechanical test on the SF-GMA hydrogels was performed, and the tensile YounG′s modulus and toughness were calculated from the acquired tensile stress–strain curves (Figure 4C,D and Figure S2D). The YounG′s modulus of the photo-crosslinked hydrogel after directional freezing is higher than that of the control group, which has YounG′s modulus of about 7.9 kPa. The tensile mechanical properties of the SF hydrogels increase steadily, reaching 39.5 kPa in the −120 °C group, which is about four times that of the control group. The tensile strength also exhibits a similar pattern, with 6.8 kPa and 53.1 kPa for the control group and the −120 °C group, respectively. There is no substantial difference in the elongation at the break between these groups, so the toughness also follows the same trend. The toughness of the directional-freezing groups is higher than that of the control group and the lower the freezing temperature, the greater the toughness. The space between the lamellar structures becomes tighter, and the pore size is smaller and more uniform as the temperature of the cold plate declines, further improving the mechanical properties of the SF-GMA hydrogel. These results indicate that the introduction of directional freezing has no effect on the elongation at the break of the SF-GMA hydrogel, and it can significantly boost the strength and toughness of SF hydrogels.

To visually investigate the toughness of the hydrogel, we placed a blade on the surface of the hydrogel and pressed a 1 kg weight on it for 1 min (Figure 4B). After cutting, the DF-SF-GMA hydrogel at −60 °C maintained good integrity and no evident incision was visible. The −30 °C and −120 °C groups also exhibited the same characteristics, but the control group was easily cut off. These results further indicate that the technique reported in this work can significantly enhance the toughness of the SF-GMA hydrogel.

### 2.4. Viscoelastic Properties of SF-GMA Hydrogels

**Figure 5:**
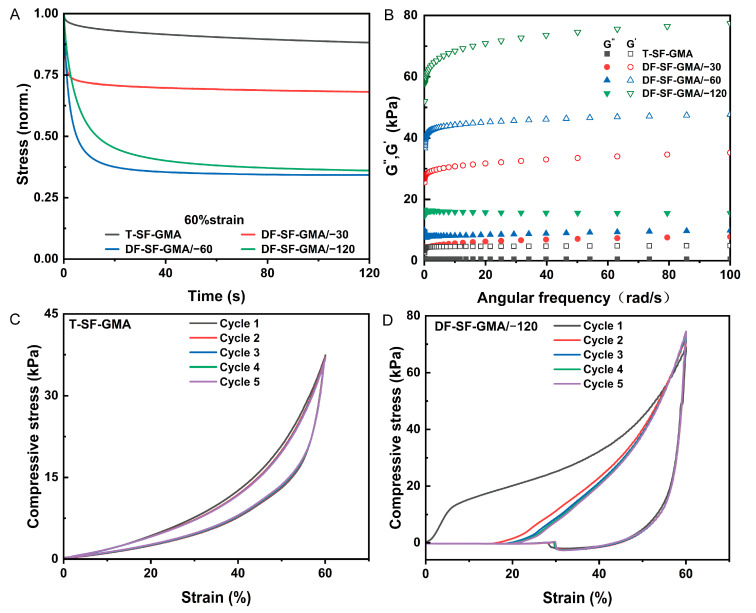
Viscoelastic properties of the T-SF-GMA and DF-SF-GMA hydrogels. (**A**) Stress relaxation test of the T-SF-GMA and DF-SF-GMA hydrogels at 60% strain. Stress is normalized by the initial stress. (**B**) Storage modulus and loss modulus of the T-SF-GMA and DF-SF-GMA hydrogels were tested using a rheometer at different frequencies. Cyclic compression test of the T-SF-GMA (**C**) and DF-SF-GMA/−120 (**D**) hydrogels under 60% strain.

Viscoelastic properties of hydrogel scaffolds have drawn much attention recently because many studies have proved that not only the stiffness of the surrounding matrix but also the viscoelastic mechanical properties, such as stress relaxation, can affect cell spreading, proliferation, and differentiation. Therefore, the viscoelastic properties of these novel scaffolds were also investigated. The stress relaxation tests were performed under 15%, 30%, and 60% strain (Figure 5A and Figure S3A–C). The results of all the groups follow the same tendency. The T-SF-GMA hydrogel possesses the slowest stress relaxation speed, indicating high elasticity, which is consistent with previous studies. The hydrogels with aligned porous structures exhibit fast stress relaxation compared to the control group. Moreover, the degree of stress relaxation rises with decreasing freezing temperature under the same strain. The stress relaxation of the −60 °C and −120 °C groups could exceed 50% under the strain of 60%, and the time required for the −120 °C group to relax to 50% stress is around 5 s. These findings indicate that, with the introduction of directional freezing, the SF-GMA hydrogel has tunable viscoelasticity, which may be beneficial for cell culture and tissue regeneration.

Rheological analysis of the SF-GMA hydrogels was also performed to explore their viscoelasticity (Figure 5B). The loss modulus G″ and the storage modulus G′ of the directional-freezing group are much larger than those of the control group, and the loss modulus G″ and the storage modulus G′ rise as the temperature of the freezing plate decreases. These results further reveal that the introduction of directional freezing enhances the viscoelasticity of the SF-GMA hydrogels, with the augmentation being more pronounced at lower freezing temperatures.

To evaluate the effect of cyclic loading and the viscoelastic properties of the scaffolds, a cyclic compression test was also conducted (Figure 5C,D and Figure S3D). The results show that the ratio of the hysteresis loop area of the traditional hydrogel is relatively small, and all the compression cycles essentially overlap, indicating the elasticity of the T-SF-GMA hydrogel. In the directional-freezing group, the proportion of the hysteretic area increases as the temperature of the cold plate decreases. The hysteretic area of the first compression cycle is larger than that of the last four compression cycles. This may be due to the fact that the oriented structure produced by directional freezing is crushed and water is squeezed out during the first compression cycle. Interestingly, there is a sudden change of stress in the DF-SF-GMA/−120 group, which could be attributed to the adhesion force of water and compression plates during the force unloading. These results illustrate that the DF-SF-GMA hydrogels display special architectures and tunable viscoelastic properties when compared to the traditional SF-GMA hydrogels.

**Figure 6:**
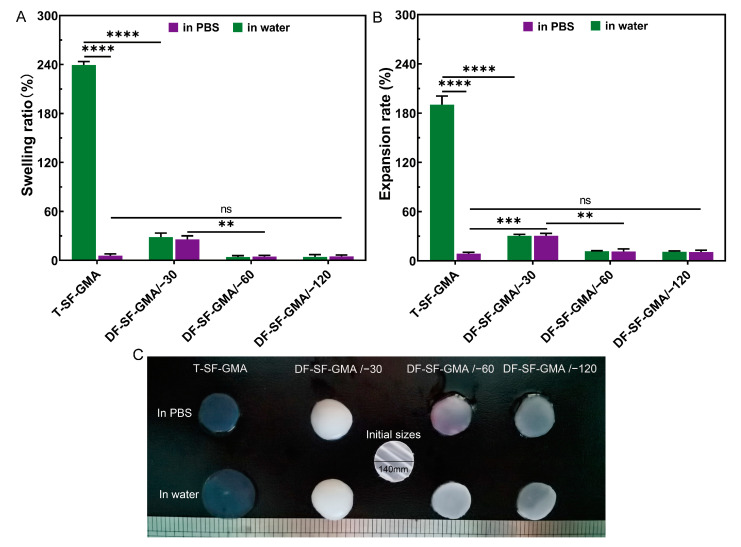
The (**A**) swelling ratio and (**B**) expansion rate of the T-SF-GMA and DF-SF-GMA hydrogels in PBS and milli-Q water. (**C**) Photomicrographs of all the hydrogels after swelling for 24 h (** *p* < 0.01; *** *p* < 0.001; and **** *p* < 0.0001, *n* = 4).

The swelling of hydrogels is also an important concern for tissue regeneration applications, depending on the circumstances. The swelling and expansion ratios of the SF-GMA hydrogels were tested in water and PBS, respectively (Figure 6). The swelling ratio of the T-SF-GMA hydrogel is 240% in water and only about 6% in PBS. Interestingly, the −30 °C, −60 °C, and −120 °C groups show similar swelling and expansion ratios in water and PBS. The main reason for this behavior is that the SF-GMA solution concentration increased and resulted in a higher crosslinking density during the DF. Therefore, the swelling ratio and expansion degree of the DF-SF-GMA hydrogels are nearly the same whether in water or in the PBS solution. The swelling and expansion degree of the −30 °C group is slightly higher than that of the −60 °C and −120 °C groups, which may be affected by the crosslinking density and the large pore size. These findings suggest that the introduction of directional freezing could strengthen the swelling resistance of SF-GMA hydrogels.

### 2.5. Cell Biocompatibility

**Figure 7:**
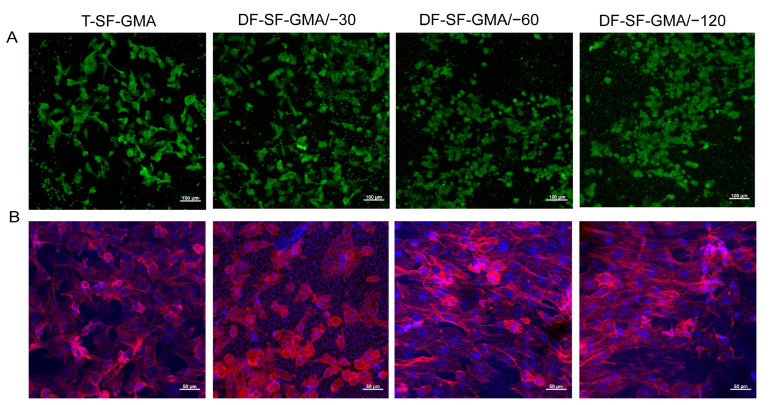
Cytocompatibility of the SF-GMA hydrogels. (**A**) Live/dead staining (Green: live cells; red: dead cells) and (**B**) F-actin staining (Blue: nucleus; red: F-actin) of cells cultured in the T-SF-GMA and DF-SF-GMA hydrogels for 3 days. All the cells exhibit excellent viabilities, and the cells cultured on the DF-SF-GMA/−60 and DF-SF-GMA/−120 hydrogels are aligned due to the surface morphology.

To study the biocompatibility of the DF-SF-GMA hydrogel scaffolds and assess their potential for clinical usage in the future, we conducted in vitro cell culture experiments. Live/dead and cytoskeleton staining was performed after the cultivation of the cells in the scaffolds for three days. The cells in all groups maintain good viability, with nearly no dead cells seen (Figure 7A). It was found that the cell spreading in the −60 °C and −120 °C groups is better than that in the −30 °C and the control groups, which may be due to the various mechanical properties of the SF-GMA (Figure 7B). Meanwhile, a considerable degree of cell orientation is generated in the −60 °C and −120 °C groups, which is induced by the lamellar structure on the surface of the DF-SF-GMA hydrogels.

It has been proved that an orientated morphology can favor the growth and differentiation of some particular cells, such as endothelial cells [49], nerve [50], and muscle cells [51], and thus achieve a better outcome in tissue regeneration. Therefore, numerous studies have constructed scaffolds with oriented structures on the surface to stimulate cell alignment and tissue regeneration. For example, aligned electrospun fibers are employed to guide vascular endothelial cell alignment for vascular tissue regeneration [49]. Similarly, collagen scaffolds with a microgroove structure are created using liquid dispensing and freeze drying to mimic muscle basement membrane [32]. However, the majority of these research studies create oriented structures on the surface of scaffolds rather than inside three-dimensional scaffolds. Recently, an internal orientated and anisotropic structure inside a hydrogel is achieved using directional freezing technology, and the encapsulated cells show a high viability when combined with cryoprotective agents [52]. Although we have only so far cultivated cells on the surface of hydrogel scaffolds, cells could be encapsulated in the DF-SF-GMA hydrogels with cryoprotectants and employed for in vitro cell culture and in vivo tissue regeneration.

These results demonstrate the good biocompatibility of the DF-SF-GMA hydrogels produced via directional freezing and photo-crosslinking. Additionally, their tunable mechanical and structural properties can serve other functions of regulating cell behaviors and promoting tissue regeneration. Furthermore, this technology can be used not only for the construction of SF scaffolds but also for the manufacture of other material-based photo-crosslinking hydrogels.

## 3. Conclusions

In this study, DF-SF-GMA hydrogel for tissue-engineering scaffold is developed by directional freezing and in situ photopolymerization. The secondary structure, microstructure, mechanical properties, and biocompatibility of the DF-SF-GMA hydrogels are investigated. The results confirm that directional freezing at different temperatures can produce SF-GMA hydrogels with oriented lamellar structures. Directional freezing reduces the content of β-sheet and increases the content of α-helix and β-turn. The mechanical properties of the SF-GMA hydrogels can be tuned by adjusting the temperature of the cold plate for directional freezing. The prepared hydrogels outperform traditional hydrogels in mechanical strength and toughness. In addition, the DF-SF-GMA hydrogels have unique viscoelasticity and anti-swelling capability. Furthermore, these DF-SF-GMA hydrogel scaffolds also exhibit excellent biocompatibility. This work provides a new method for the preparation of hydrogels with aligned porous structures, which is expected to broaden the biomedical applications of hydrogels.

## 4. Materials and Methods

### 4.1. Reagents and Materials

Silkworm cocoons were obtained from the Northwest Silkworm Base (Shanxi, China). Lithium bromide (LiBr), sodium carbonate (Na_2_CO_3_), glycidyl methacrylate (GMA), and photoinitiator (Irgacure2959) were purchased from Aladdin (Aladdin, Shanghai, China). All reagents were used as received.

### 4.2. Synthesis of SF-GMA Macromer and ^1^H NMR

The SF-GMA was synthesized by GMA modification according to a previous method. Briefly, the cocoons were boiled in a 0.05 M Na_2_CO_3_ solution for 30 min to remove sericin, and then they were washed several times with distilled water. Subsequently, 30 g of degummed and dried silk fibroin was dissolved in 150 mL of 9.3 M LiBr solution at 60 °C for 1 h, after which 9 mL of GMA solution was added to the mixture and stirred at 300 rpm for 3 h. The resulting solution was then dialyzed against milli-Q water for 4 days. Finally, the obtained methacrylate SF solution was concentrated to 20% using a 3500 Da dialysis bag against a PEG solution with a molecular weight of 8000.

The synthesis of the SF-GMA macromer was confirmed using ^1^H NMR. After lyophilization, 5 mg of the regenerated silk fibroin (RSF) and SF-GMA was dissolved in 700 μL of deuterium oxide. The ^1^H NMR spectra were collected using a Bruker DPX FT-NMR spectrometer with a frequency of 400 MHz. The degree of methacrylate substitution was determined using the formula of 1-(lysine integration signal of SF-GMA/lysine integration signal of unsubstituted RSF).

### 4.3. Preparation of SF-GMA Hydrogel

Irgacure2959 (0.5 *w*/*v*%) was added into the above-mentioned SF-GMA solution, and the solution was stirred slowly at 45 °C for one hour. As shown in Figure 1, the solution was directionally frozen on copper plates with different temperatures of −30 °C, −60 °C, and −120 °C. During directional freezing, an oriented lamellar porous structure was created [53,54]. After complete freezing, the quartz glass plate was covered on the scaffold, and they were subjected to ultraviolet light for 8 min. The solution was melted and crosslinked simultaneously during UV exposure, yielding directional-freezing, photo-crosslinked silk hydrogels (DF-SF-GMA). The bulk traditional silk hydrogel (T-SF-GMA), as the control group, was produced by direct photo-crosslinking for 8 min without directional freezing.

### 4.4. Structure Characterization

To analyze the internal microstructure of the silk fibroin hydrogels, the T-SF-GMA and the DF-SF-GMA hydrogels were observed using an SEM at a voltage of 15 kV. The prepared DF-SF-GMA and T-SF-GMA hydrogel samples were frozen at −80 °C and then lyophilized. After which, the samples were cut along the freezing direction, and the sections were coated in platinum using a sputtering system (SC7620, Quorum, San Diego, CA, USA).

### 4.5. FTIR Analysis

The hydrogels were ground with KBr after freeze drying to prepare the sample for Fourier-transform infrared spectrometer (FTIR, Thermo Scientific Nicolet iS20, Waltham, MA, USA). The spectra were recorded over the wavenumber range from 400 to 4000 cm^−1^ with a resolution of 4 cm^−1^. Fourier self-deconvolution (FSD) of the infrared spectra covering the amide I at 1600−1700 cm^−1^ was performed using the PeakFit software. The FSD Spectra were curve-fitted to explore the relative areas of the amide I region components and the secondary structure of SF.

### 4.6. Mechanical Characteristics

To assess the mechanical properties of the hydrogels under physiological conditions, the DF-SF-GMA and T-SF-GMA hydrogels were punched into a disk with a diameter of 6 mm and a height of 2 mm and then swollen in a PBS solution at 37 °C for 24 h before the following mechanical testing (*n* = 3). For the compressive strain–stress test, the samples were subjected to a compression test at a rate of 1 mm/min. For the stress relaxation test, all samples were first compressed at a strain rate of 1 mm/min. The strain was held at 15%, 30%, and 60% strain, and the relaxation time was recorded to compare the stress relaxation of the hydrogels [55].

To measure the tensile properties, the hydrogel samples were cut into a rectangular shape and were tested at a strain rate of 3 mm/min. The toughness of the hydrogels was calculated according to the area of the tensile stress–strain curve. The rheological properties of the hydrogels were measured using a rheometer (TA Discovery DHR, Delaware, DE, USA) at 37°C, with an angular frequency sweep range of 0.1 to 100 rad/s and a shear strain of 1%.

### 4.7. Swelling Ratio Measurement

To test the swelling ratio, the hydrogels were swollen in PBS or water at 37 °C for 24 h. The weights *m*_1_ and *v*_1_ before swelling were recorded. After swelling, the samples were blotted with a filter paper to remove the residual water and weighted (*m*_2_ and *v*_2_). The swelling ratio *Q* and the swelling degree *E* were calculated according to the following formulas (*n* = 4):

$$Q=\frac{m_{2}-m_{1}}{m_{1}}\times 100\%$$

$$E=\frac{v_{2}-v_{1}}{v_{1}}\times 100\%$$

### 4.8. Biocompatibility

Human umbilical vein endothelial cells (HUVEC) were employed as the cell models to verify the biocompatibility of the DF-SF-GMA hydrogels. The HUVEC were cultured in 75 cm^2^ tissue culture flasks in a culture medium (DMEM, Biological Industries Ltd., Shanghai, China.) supplemented with 10% fetal bovine serum (FBS, Biological Industries, Shanghai, China), 100 U mL^−1^ penicillin (Solarbio, Beijing, China), and 100 μg mL^−1^ streptomycin (Solarbio, Beijing, China) at 37 °C and 5% CO_2_. The cell culture medium was refreshed every two days. After reaching 90% confluence, the cells were detached, suspended, and seeded into the hydrogels. Before cell seeding, the hydrogels were cut into small disks and sterilized in a 24-well plate. After incubation for 3 days, cell viability and cytoskeleton staining were performed using the live/dead kit and phalloidin staining. The fluorescence images were captured using a confocal microscope (LSM 880, Zeiss, Freiburg, Germany).

### 4.9. Statistical Analysis

All experiments were conducted independently three times with 3~6 samples to calculate the mean ± standard deviation (SD). The image data were processed using ImageJ. Statistical analysis was performed using Prism 6.0 (GraphPad Software, San Diego, CA, USA) and Origin Pro to evaluate the significance of the experimental data, and *p*-values less than 0.05 were considered statistically significant.

## Data Availability

Not applicable.

## Supplementary Materials

The following supporting information can be downloaded at: https://www.mdpi.com/article/10.3390/gels9030181/s1, Figure S1. The deconvolution of FTIR spectra for the amide I band for (A) T-SF-GMA and (B) DF-SF-GMA/−120 hydrogels; Figure S2. Physical properties of DF-SF-GMA hydrogels. The compressive stress-strain curves at early (A) and late stage (B). (C) The compressive modulus of all the hydrogels at different strains. The compressive modulus of DF-SF-GMA/−60 and DF-SF-GMA/−120 were much higher than other groups. (D) Tensile elastic modulus of T-SF-GMA and DF-SF-GMA hydrogels (* *p* < 0.05; ** *p* < 0.01; *** *p* < 0.001; and **** *p* < 0.0001, *n* = 3); Figure S3. Viscoelastic properties of T-SF-GMA and DF-SF-GMA hydrogels. Stress relaxation test of T-SF-GMA and DF-SF-GMA hydrogels at a strain of (A) 15% and (B) 30%. Stress is normalized by the initial stress. (C) The ratio of relaxed stress to initial stress at 60 s. (D) Cyclic compression test of DF-SF-GMA/−30 hydrogels under 60% strain.
