# Supplementary material for: Fabrication of Silk Hydrogel Scaffolds with Aligned Porous Structures and Tunable Mechanical Properties
Source: Gels. 2023 Feb 24;9(3):181. doi: 10.3390/gels9030181 (PMC10048404; doi:10.3390/gels9030181)
Supplement: Supplementary file 1 [file gels-09-00181-s001.zip › gels-2240446-supplementary.pdf]

Supplementary Information for

Fabrication of Silk Hydrogel Scaffolds with Aligned Porous Structures and Tunable Mechanical Properties

Zewu Jiang[1,2], Qingqing Sun[3], Qian Li[1,2*] and Xiaomeng Li[1,2*]

[1] School of Mechanics and Safety Engineering, Zhengzhou University, Zhengzhou 450001, China
[2] National Center for International Joint Research of Micro-nano Moulding Technology, Zhengzhou University, Zhengzhou 450001, China
[3] School of Materials Science and Engineering, Zhengzhou University, Zhengzhou 450001, China

* Correspondence: xiaomeng.li@zzu.edu.cn; qianli@zzu.edu.cn

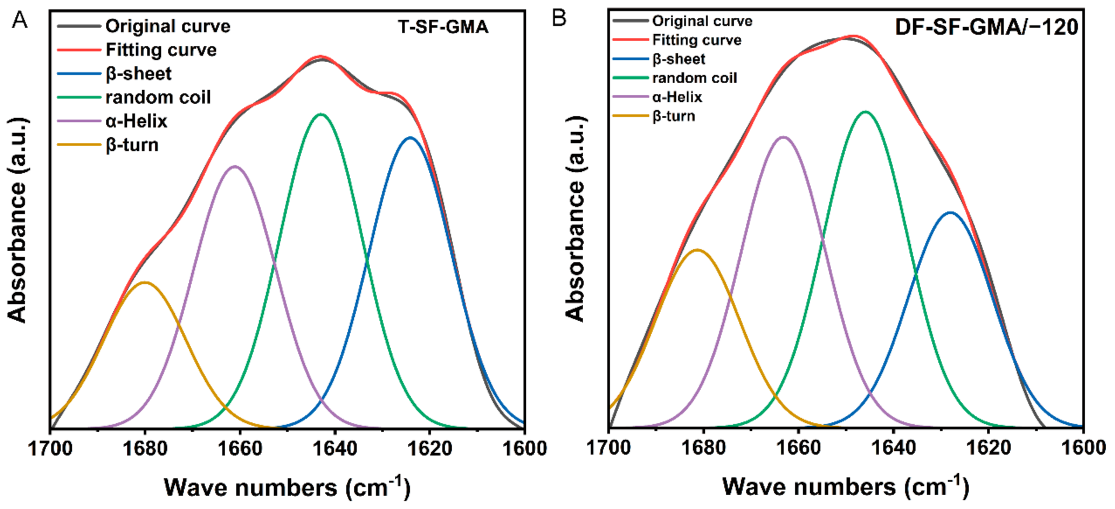

Figure S1. The deconvolution of FTIR spectra for the amide I band for (A) T-SF-GMA and (B) DF-SF-GMA/−120 hydrogels.

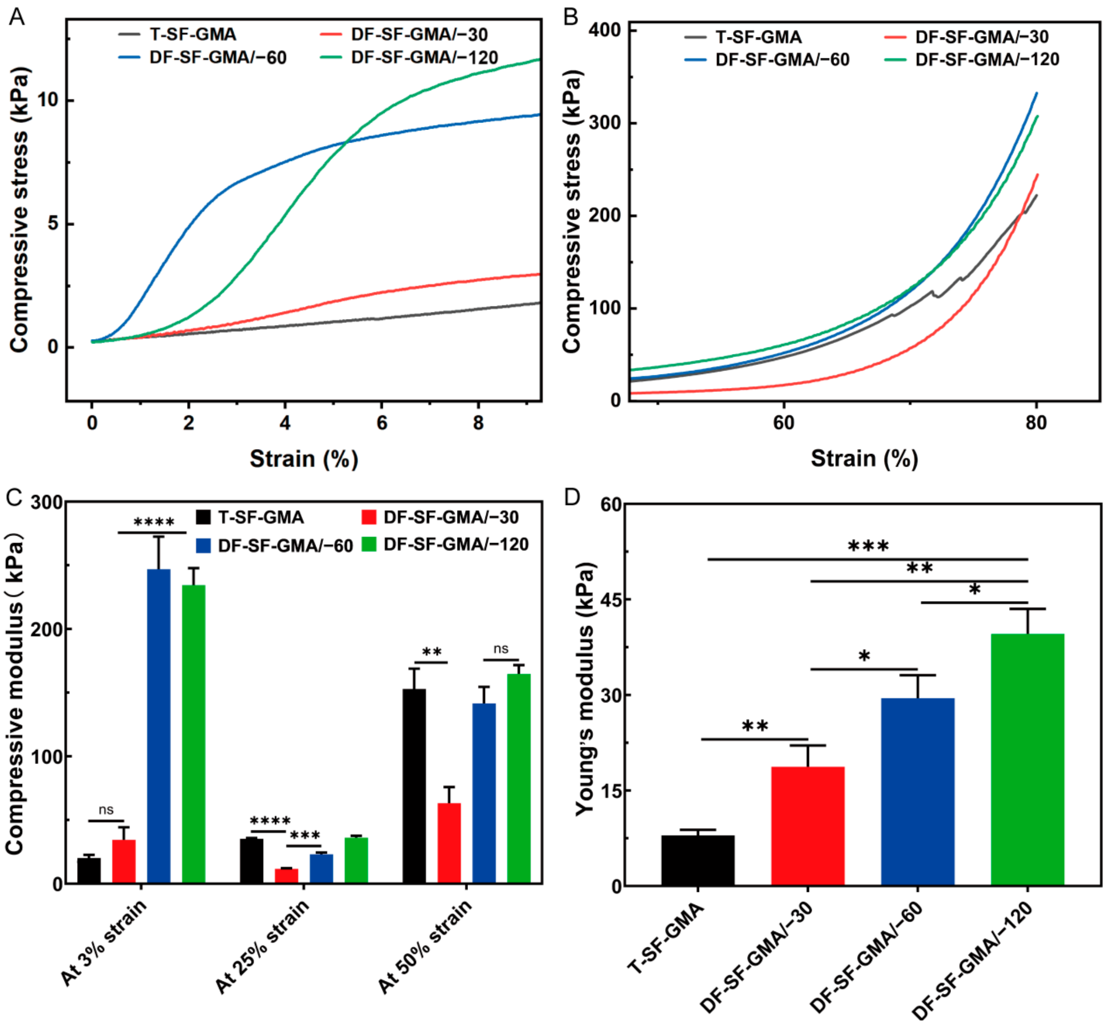

Figure S2. Physical properties of DF-SF-GMA hydrogels. The compressive stress-strain curves at early (A) and late stage (B). (C) The compressive modulus of all the hydrogels at different strains. The compressive modulus of DF-SF-GMA/−60 and DF-SF-GMA/−120 were much

higher than other groups. (D) Tensile elastic modulus of T-SF-GMA and DF-SF-GMA hydrogels (* $P < 0.05$; ** $P < 0.01$; *** $P < 0.001$; and **** $P<0.0001$, n = 3).

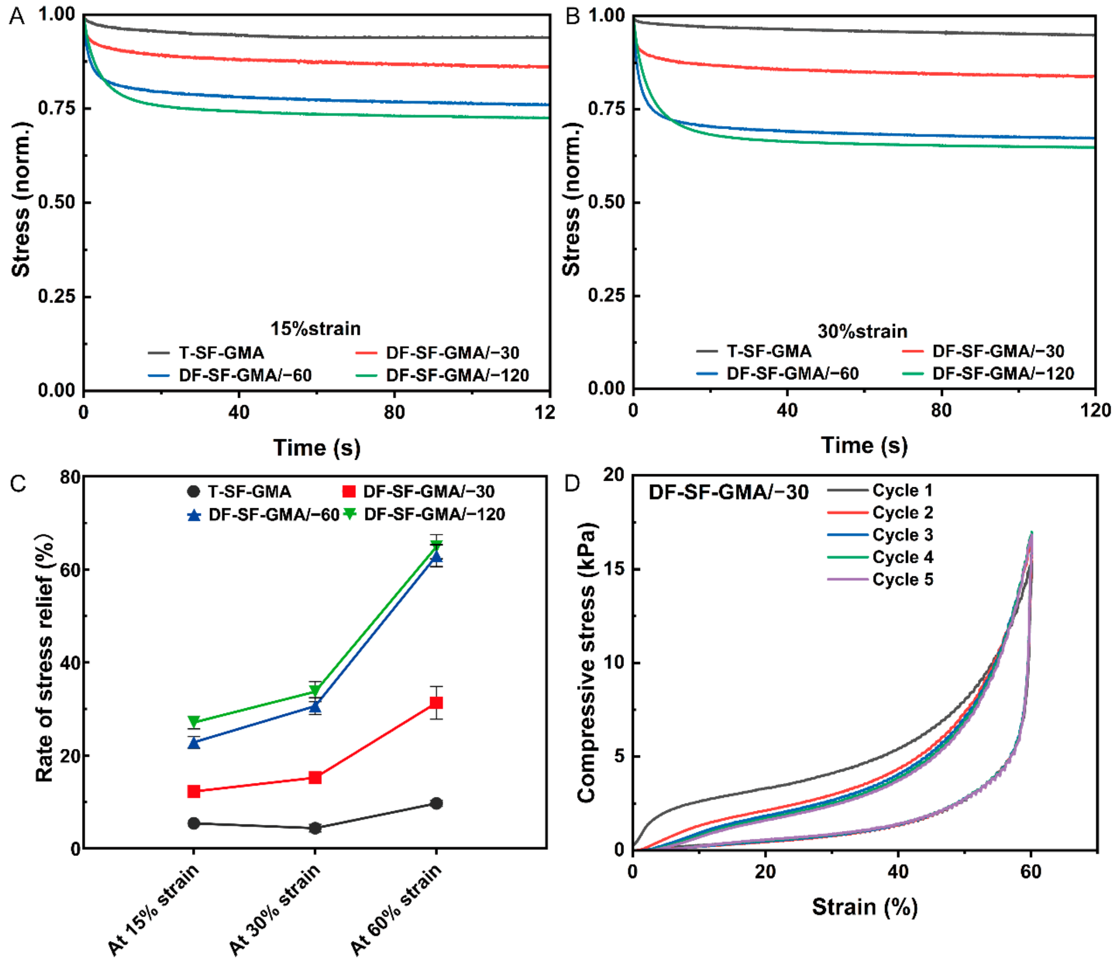

Figure S3. Viscoelastic properties of T-SF-GMA and DF-SF-GMA hydrogels. Stress relaxation test of T-SF-GMA and DF-SF-GMA hydrogels at a strain of (A) 15% and (B) 30%. Stress is normalized by the initial stress. (C) The ratio of relaxed stress to initial stress at 60 s. (D) Cyclic compression test of DF-SF-GMA/–30 hydrogels under 60% strain.
